# A brief review of exercise, bipolar disorder, and mechanistic pathways

**DOI:** 10.3389/fpsyg.2015.00147

**Published:** 2015-03-04

**Authors:** Daniel Thomson, Alyna Turner, Sue Lauder, Margaret E. Gigler, Lesley Berk, Ajeet B. Singh, Julie A. Pasco, Michael Berk, Louisa Sylvia

**Affiliations:** ^1^Department of Applied Sciences, Royal Melbourne Institute of Technology University, Bundoora, VIC, Australia; ^2^Innovation in Mental and Physical Health and Clinical Treatment Strategic Research Centre, School of Medicine, Deakin University, Geelong, VIC, Australia; ^3^Department of Psychiatry, University of Melbourne, Parkville, VIC, Australia; ^4^Centre for Translational Neuroscience and Mental Health, School of Medicine and Public Health, University of Newcastle, Callaghan, NSW, Australia; ^5^Federation University Australia, Ballarat, VIC, Australia; ^6^Department of Psychiatry, Massachusetts General Hospital, Boston, MA, USA; ^7^Mental Health and Wellbeing Strategic Research Centre, School of Psychology, Deakin University, Geelong, VIC, Australia; ^8^Department of Medicine, NorthWest Academic Centre, University of Melbourne, St Albans, VIC, Australia; ^9^Florey Institute for Neuroscience and Mental Health, Parkville, VIC, Australia; ^10^Orygen, The National Centre of Excellence in Youth Mental Health, Parkville, VIC, Australia; ^11^Harvard Medical School, Harvard University, Boston, MA, USA

**Keywords:** bipolar disorder, exercise, mechanistic pathways, depression, hypomania, neurogenesis

## Abstract

Despite evidence that exercise has been found to be effective in the treatment of depression, it is unclear whether these data can be extrapolated to bipolar disorder. Available evidence for bipolar disorder is scant, with no existing randomized controlled trials having tested the impact of exercise on depressive, manic or hypomanic symptomatology. Although exercise is often recommended in bipolar disorder, this is based on extrapolation from the unipolar literature, theory and clinical expertise and not empirical evidence. In addition, there are currently no available empirical data on program variables, with practical implications on frequency, intensity and type of exercise derived from unipolar depression studies. The aim of the current paper is to explore the relationship between exercise and bipolar disorder and potential mechanistic pathways. Given the high rate of medical co-morbidities experienced by people with bipolar disorder, it is possible that exercise is a potentially useful and important intervention with regard to general health benefits; however, further research is required to elucidate the impact of exercise on mood symptomology.

## BACKGROUND

Bipolar disorder is a chronic condition characterized by elevated (manic) and depressive episodes often associated with difficulty functioning and poor quality of life. A diagnosis of bipolar disorder is also associated with an increased risk of cardiovascular disease leading to premature mortality (Roshanaei-Moghaddam and Katon, 2009; Dome et al., 2012; Crump et al., 2013). Further, obesity and a sedentary lifestyle are risk factors for diabetes, metabolic syndrome and cardiovascular disease, all of which disproportionally affect people with bipolar disorder (Elmslie et al., 2001; Morriss and Mohammed, 2005; Alsuwaidan et al., 2009; Cairney et al., 2009; Kilbourne et al., 2009). Thus, individuals with bipolar disorder face the dual struggle of needing to focus their attention and treatment on not only their mental health but also their physical health.

Exercise may be an excellent candidate to meet this need. Exercise unequivocally improves physical heath (e.g., obesity, cardiorespiratory fitness, blood pressure, cholesterol; Cornelissen and Fagard, 2005; Church et al., 2007; Department of Health, 2011), but recent data also suggest that exercise is an effective treatment of depression and anxiety (Daley, 2008; Wipfli et al., 2008; Rethorst et al., 2009; Moylan et al., 2013; Rethorst and Trivedi, 2013). These data have prompted some to view exercise as a first line of treatment for mild to moderate depression (Carek et al., 2011). Given the promising data for depression and anxiety, exercise may also prove to be beneficial for the management of bipolar disorder. Specifically, evidence suggests that exercise is neuroprotective at least in part by increasing brain derived neurotrophic factor (BDNF; Sylvia et al., 2010). Other mechanisms will be explored, including the genetic expression and endorphin hypothesis.

The aim of this review is to understand the amount of exercise and physical activity currently engaged in by individuals with bipolar disorder. For the purpose of this review, exercise is defined as a conscious, planned decision to move and be physically active, whereas physical activity refers to any movement, including leisure activity, occupational activity, or other activities of daily living (Caspersen et al., 1985; Thompson et al., 2003). A second aim is to evaluate the research on the role of exercise in improving physical (obesity, blood pressure) and mental (symptoms, quality of life) health outcomes in bipolar disorder. Finally, we will discuss the potential mechanisms of how exercise is suspected of improving mood and functioning in bipolar disorder.

## METHODS

**FIGURE 1:**
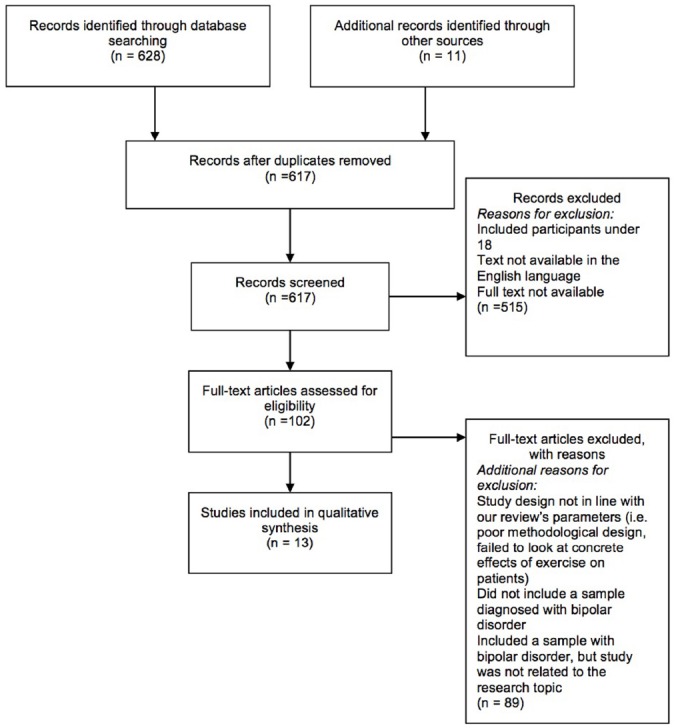
**Consort flowchart for study selection**.

We conducted our search using Google Scholar, Proquest, CINAHL Complete, PubMed, and PSYCINFO, including unpublished papers in the form of dissertation abstracts using the search terms bipolar disorder and exercise and bipolar disorder and physical activity. Based on this initial search, we found 628 articles. We conducted two independent reviews of the literature by two separate authors (DT and MEG). The review period was September through November, 2014. We found over 600 articles when searching for “bipolar disorder” and “exercise” or “bipolar disorder” and “physical activity.” We then limited this search by only using studies that focused on adult participants with a diagnosis of bipolar disorder; studies that included participants below the age of 18 were excluded, as were studies that were not in the English language. Studies were also excluded if they did not explicitly focus on the effects of exercise on patients with mental illness or if they did not relate directly to the review topic (i.e., a study that monitored activity levels on patients via their smartphones saw lower levels of activity, but was merely correlational; Faurholt-Jepsen et al., 2014). Thus, 13 studies are included in this review (see Figure 1, Table 1).

**TABLE 1:** **Summary of reported studies and their characteristics**.

**Author**	**Study design**	**Participants**	**Intervention**	**Results**	**Methodological quality**
Elmslie et al. (2001)	Cross-sectional	89 participants with bipolar disorder, all of whom were currently in outpatient treatment psychiatric diagnoses were based on ICD-9 codes	Main outcome measures included macronutrient intakes, percentage of energy derived from food sources and physical activity levels	Mean total energy intake was higher in female patients than reference subjects Patients also reported lower frequencies of physical activity compared to the reference subjects	Participants’ data were collected from a VA’s office, which may not be representative of the general population
Ng et al. (2007)	Retrospective cohort study	Admissions to inpatient unit with primary diagnosis of ICD-10 bipolar disorder (*N* = 98 admissions across 49 patients), 15 males	Participation in walking group—40 min walking intensity determined by participants up five times weekly Comparison—non-participating patients	The two groups did not differ significantly in demographics or admission clinical global impression (CGI) and depression anxiety stress scale (DASS) measures, except for a lower DASS stress subscore for participants (*p* = 0.049) than non-participants (*p* = 0.005) and all its subscales (Depression *p* = 0.048, Anxiety *p* = 0.002, Stress *p* = 0.01)	Retrospective design, small sample size, lack of randomization or control, and indirect measure of manic symptoms
Kilbourne et al. (2007)	Cross-sectional	Patients who completed the VA’s Large Health Survey of Veteran Enrollees section on health and nutrition in 1999 and who received a diagnosis of bipolar disorder (BD) (*n* = 2032), schizophrenia (*n* = 1895) or were included in a random sample of non-SMI VA patients (*n* = 3065)	Authors compared nutrition and exercise behaviors using multivariate logistic regression, controlling for patients socioeconomic status (SES) and clinical factors and adjusting for patients clustered by sites using generalized estimating equations	Patients with BD were more likely to report poor exercise habits, including infrequent walking or strength exercises compared to those with no standardised mortality index (SMI)	The nature of the data was self-report
Shah et al. (2007)	Between-groups AB	*N* = 24 (14 individuals with bipolar disorder clinically assessed as euthymic, 10 controls), 14 males	Treadmill exercise at 10% gradient at 70% maximal predicted oxygen consumption. Duration until exhaustion	Exercise duration significantly shorter in BD group (*d* = 0.47). No significant between group differences in electrocardiograph (ECG) variables	More BD patients smoked (28.6 vs 0% controls) and patients tended to be heavier, (189.1 ± 29.3 vs. 165.0 ±29.5 lb, *t* = 2.0, *p* = 0.06)
Hays et al. (2008)	Within-participants AB	*N* = 26 individuals with DSM-IV bipolar I or II disorder, 13 males	Treadmill exercise for 20 min at 70% age-predicted maximal heart rate	Significant increase in dehydroepiandrosterone sulfate (DHEAS) evel post-exercise and significant increase in self-reported well being post-exercise	Most of the participants were relatively asymptomatic (87%)
Cairney et al. (2009)	Cross-sectional	Data used from the 2002 Canadian Community Health Survey, physica activity (PA) levels were compared among individuals with BD (*n* = 831) to those with major depression (*n* = 4713) and those with no identifiable mood disorder *n* = 31,834)	Using multivariate logistic regression, the independent effects of sociodemographic and clinical factors in active and inactive BD individuals stratified by relative weight status	No differences in the proportion of ndividuals characterized as active, moderately active or inactive among ndividuals with BD, major depressive disorder (MDD), or the general population	The nature of the data was self-report
Van Citters et al. (2010)	Within-group	*n* = 76, nearly three-quarters were female (*n* = 54), psychiatric diagnoses primarily included major depressive disorder (*n* = 30, 39.5%), bipolar disorder (*n* = 19, 25.0%), and schizophrenia or schizoaffective disorder (*n* = 18, 23.7%)	Participants were assigned an ndividual health mentor and over 9 months work together to set goals regarding healthier dietary decisions as well as other modules of wellness	Mental health functioning significantly mproved among participants, as did negative symptoms. Participation in the program was associated with increased exercise, vigorous activity, and leisurely walking. Participants also demonstrated a significant reduction in waist circumference	No control group
Sylvia et al. (2011)	Within-groups	After the first group (*N* = 4) had completed the treatment, it was revised, and then a second group *N* = 6) completed the revised treatment. Participants completed al of the study assessments and attended 82% of the sessions	Three treatment modules, Nutrition, Exercise, and Wellness (NEW Tx), were administered in twelve 60-min group sessions over 14 weeks	Both groups added over 100 min of weekly exercise to their baseline duration. Group 1 did not show any significant changes in any of the outcome measures. Group 2 showed improvements in their quality of ife, depressive symptoms, and weight	Small sample size, predominantly college students and a lack of a finalized treatment manual
Wright et al. (2012)	Cross-sectional	25 individuals with BD	Semi-structured interview concerning their views on the relationship between exercise and BD. The data was then subjected to qualitative analysis using an nterpretative Phenomenologica Analysis approach	Three themes emerged—regulating exercise for mood regulation, exercise as a double-edged sword, and exercise potentially bringing structure to chaos	Qualitative analyses
Daumit et al. (2013)	Between-groups	*N* = 291) who suffered from serious mental illness—including bipolar disorder (*n* = 64, 22%), as well as schizophrenia, schizoaffective disorder, and major depression	Participants took part in an 18-month behavioral weight loss ntervention. The treatment consisted of group exercise sessions as well as individualized weight-management sessions	The intervention group lost more weight than the control group, such that 37.8% of participants in the intervention group lost at east 5% of their initial weight, compared with 22.7% in the control group	
Janney et al. (2014)	Between-groups	60 adults with BD were matched 1:1 to users and non-users of mental health services by gender, closest body mass index (BMI), and age	Adult outpatients treated for BD (>18 year) wore accelerometers for seven consecutive days. Each minute epoch was assigned an activity level based on the number of counts per minute	The majority of monitoring time (78%) was classified as sedentary. Light PA accounted for 21 % and none achieved 150 min/week of moderate to vigorous activity (as is recommended by national guidelines)	
Sylvia et al. (2013a)	Within-group	482 individuals with BD (either BP or II, in accordance with DSM IV) TR (aged 18-68)	Exercise frequency in BD patients was assessed in a multi-site comparative effectiveness study that examined a second generation antipsychotic (quetiapine) versus a classic mood stabilizer (lithium)	Approximately 40% of participants reported not exercising regularly. Less frequent exercise was associated with higher BMI, more depressive symptoms, and lower quality of life functioning. More frequent exercise was associated with experiencing more mania in the past year and more current manic symptoms	Cross sectional analysis and self-report. Intensity and state of exercise (e.g., compulsive or not compulsive) were not measured
Sylvia et al. (2013b)	Within-group	Five participants ages 23-64 years (*M* = 44). All participants had a primary diagnosis of BD as determined by a clinician-administered neuropsychiatric interview	Participants took part in NEW Tx, a 20-week individual cognitive behavioral therapy-based treatment comprising of three modules Nutrition, Exercise, and Wellness (NEW)	Participants’ weight, cholesterol, and triglycerides decreased over the study duration as well as number of daily calories and sugar intake. Weekly exercise duration more than tripled and depressive symptoms and overall functioning improved	Open trial, no control group. Smal sample size limits ability to draw stronger conclusions

## RESULTS

### PHYSICAL ACTIVITY LEVELS AND BIPOLAR DISORDER

We found 13 empirical studies that have examined the physical activity levels of individuals with bipolar disorder. In a sample of 60 outpatient adults with bipolar disorder, Janney et al. (2014) found that 78% of the 17 h day that participants wore their actigraphs was classified as sedentary (13.5 h per day) and that no participants achieved 150 min per week of moderate/vigorous exercise as recommended by UK national guidelines (National Institute for Health and Clinical Excellence, 2006). These findings are consistent with several other reports of high rates of physical inactivity in people with bipolar disorder (Elmslie et al., 2001; Kilbourne et al., 2007; Shah et al., 2007). These data are limited as the Elmslie et al. (2001) study only included patients that were currently euthymic and Kilbourne et al. (2007) despite having a large sample (*N* = 2032), utilized only a veteran population and included individuals with schizophrenia and did not have data on bipolar subtype or mood state.

Overall, physical activity levels in bipolar disorder appear to be lower than that of the general population (Elmslie et al., 2001; Janney et al., 2014), but the data are not conclusive. For example, a national survey in Canada found no significant differences in physical activity between people with and without bipolar disorder (Cairney et al., 2009). However, this study was limited by the use of self-report measures and the assessment of leisure-time physical activity only. Moreover, methodological variations, particularly with regard to the method of assessment of physical activity, make it difficult to compare across studies. In sum, given the many factors that negatively impact physical activity in bipolar disorder, such as higher rates of smoking, obesity, and medication side effects, it is not surprising that the data suggest that they are more likely to have sedentary lifestyles (Williams et al., 2009; Dodd et al., 2010; Vancampfort et al., 2013).

### ADJUNCT PHYSICAL ACTIVITY AND BIPOLAR DISORDER

Reported studies are summarized in Table 1. Very few studies have examined the potential therapeutic effects of physical activity on bipolar disorder. Ng, Dodd, and Berk invited inpatients to participate in a walking group and found that it was associated with improvements in the domains of depression, anxiety and stress (Ng et al., 2007). Despite several limitations of this study, including small sample size, lack of control for confounding variables, open nature, and no specific measure for mania, the study provides useful preliminary data in establishing exercise as a viable treatment option for patients with bipolar disorder.

In a study conducted by Hays et al. (2008), 26 patients diagnosed with either Bipolar I or Bipolar II Disorder walked or ran on a treadmill for 20 min at 70% of their age-predicted maximal heart rate. Findings revealed significant increases in self-reported well-being and the hormone dehydroepiandrosterone sulfate, a precursor of the adrenal steroid dehydroepiandrosterone (Wright et al., 2009). Although levels of dehydroepiandrosterone and overall well-being improved over the study duration, there was no significant correlation between the two variables (Hays et al., 2008).

Daumit et al. (2013) recruited participants (*N* = 291) who suffered from serious mental illness—including bipolar disorder (*n* = 64, 22%), as well as schizophrenia, schizoaffective disorder, and major depression—to participate in an 18-month behavioral weight-loss intervention. The treatment consisted of group exercise sessions as well as individualized weight-management sessions. The intervention group lost more weight than the control group, such that 37.8% of participants in the intervention group lost at least 5% of their initial weight, compared with 22.7% in the control group. These findings show that overweight and obese individuals with serious mental illness are capable of implementing lifestyle changes taught by an intervention, despite the daily difficulties posed by their illness.

In another community-integrated program, Van Citters et al. (2010) developed a manual for an intervention program known as “In SHAPE,” a lifestyle intervention manual for patients with serious mental illness. The pilot study included participants with schizophrenia, bipolar disorder, or major depressive disorder. Participants were assigned an individual health mentor and over 9 months work together to set goals regarding healthier dietary decisions as well as other modules of wellness. Importantly, mental health functioning significantly improved among participants, as did negative symptoms. Participation in the program was associated with increased exercise, vigorous activity, and leisurely walking. Participants also demonstrated a significant reduction in waist circumference.

Sylvia et al. (2011) developed an integrated psychosocial treatment, or the Nutrition, Exercise, and Wellness Treatment (NEW Tx), specifically to help individuals with bipolar disorder engage in healthier lifestyle habits. NEW Tx consists of three modules to target changes related to eating more nutritiously and with better portion control, increase weekly exercise as well as improve other areas of wellness (i.e., sleep, smoking/substance use). In the NEW Tx pilot study, five participants completed the 20-week individual cognitive behavioral therapy-based treatment. Participants entering the study tended to be mildly to moderately depressed [baseline MADRS = 17.2 (5.2); baseline YMRS = 4.4 (2.0); Sylvia et al., 2013b]. Participants attended most of the NEW Tx sessions and reported high satisfaction with the treatment. Participants increased intake of vegetables and decreased their daily intake of sweets. Participants’ weight, cholesterol (total, high-density lipoprotein cholesterol and low-density lipoprotein) triglycerides, and plasma glucose declined from baseline to 20 weeks follow-up. Moreover, participants experienced improvement of depressive symptoms and overall functioning as well as tripling their amount of exercise. This is one of the first studies to demonstrate the feasibility and tolerability of an intensive lifestyle intervention for bipolar disorder with promising data for its efficacy.

These lifestyle interventions hold promise in that they demonstrate that participants with serious mental illnesses can succeed in wellness programs that have been proven successful in the general population. In order to further examine the efficacy of these programs, it is necessary to conduct more studies of this nature in randomized, controlled trials.

### PHYSICAL ACTIVITY AND MANIA

Despite the lack of literature on exercise and physical activity in bipolar disorder, there is preliminary evidence that exercise may be a double-edged sword for patients with bipolar disorder due to it potentially polar-specific effect (Wright et al., 2012; Sylvia et al., 2013a). Wright et al. (2012) conducted a semi-structured study with 25 participants diagnosed with bipolar disorder, in which participants were interviewed on their experiences with exercise and their illness. Several themes emerged, including that of the “double-edged sword” theory, or that exercise brought structure and support for some patients while not helping others. Specifically, they found that exercise could be beneficial in helping to direct excess energy, but potentially detrimental in exacerbating manic symptoms and potentially putting patients at risk for a spiraling of manic and hypomanic symptoms. The aggravation of manic symptoms could be mediated by direct effects on mood or indirectly on excessive goal striving, which has been hypothesized to be a psychological risk pathway in the disorder (Nusslock et al., 2007; Alloy et al., 2012). Interestingly, patients described that forms of exercise with an inherent rhythm may provide a somewhat calming effect and facilitate mood regulation due to the cadenced nature of activities such as walking, running, or swimming (Wright et al., 2012). Importantly, in another study conducted by Suto et al. (2010), exercise and rest were identified as being; among the most helpful factors in managing bipolar disorder, with a specific theme on finding the right type of exercise, which could be individually dependent. Although qualitative in nature, these studies highlight that components of an exercise program, including type, intensity, frequency, and duration may be particularly important to investigate when examining the relationship between exercise and bipolar disorder.

Although it has been proposed that exercise may have a double-edged effect on people with bipolar disorder, empirical evidence is needed to support this claim. In their qualitative study, Wright et al. (2012) also suggested that while some participants experienced increased activation levels following exercise, other participants found exercise to have a calming effect on hypomania while Suto et al. (2010) recognized exercise as a popular wellness strategy for patients with bipolar disorder, with a particular theme on finding the right type of exercise (Suto et al., 2010). This is a topical debate with important implications, and future studies are suggested to examine the effects of exercise during mania and hypomania including potential addiction to exercise in this population.

Similarly, Sylvia et al. (2013a) conducted a multi-site comparative study of a second generation antipsychotic (quetiapine) versus a classic mood stabilizer (lithium) in a cohort of 482 people with bipolar disorder. Importantly, individuals in a manic, hypomanic or mixed state at study entry tended to exercise at a greater frequency than currently depressed individuals. These data further support that there may be a complex relationship between bipolar disorder and exercise, although it was unclear if their mood was driving the exercise behavior, or if there was a bidirectional relationship. The authors suggested a specific relationship between exercise frequency and mood polarity, such that depression is associated with less exercise and mania with more exercise in people with bipolar disorder. While the association of increased energy and activity with mania, and its converse with depression, may simply be an illustration of the core symptomatology of the disorder, another explanation for this polar-specific relationship could be the behavioral activation system (BAS; Meyer et al., 2007; Proudfoot et al., 2012; Wright et al., 2012). The BAS, a neurobehavioural system thought to regulate behavior in response to incentives and reward, is thought to be hyper-responsive in individuals with bipolar disorder. While depressive symptoms may emerge following a failure to achieve, or loss of goals/reward (BAS deactivation), hypomania or mania may be triggered in vulnerable individuals following a BAS activation event (an opportunity to gain a desired reward/goal; Urosevic et al., 2008). Individuals who are prone to hypomania or mania; therefore, may be more likely to pursue potentially pleasurable activities with greater vigor and enthusiasm due to the increased responsivity of this reward system (Meyer et al., 2007). Exercise could be considered a goal striving activity, explaining why some people demonstrate an addiction-like tendency to over exercise during a manic episode (Meyer et al., 2007; Wright et al., 2012).

In sum, the relationship of physical activity and mania is still unclear. For example, regular physical activity is associated with better sleep quality in individuals with bipolar disorder (Nusslock et al., 2007; Wright et al., 2012), and meta-analytical reviews have noted that exercise results in increased total sleep, increased slow wave sleep and decreased REM sleep (Kubitz et al., 1996; Youngstedt et al., 1997). Given that sleep problems are a prodromal symptom of mania, physical activity may still have some benefit just before and during a manic phase (Ng et al., 2007; Suto et al., 2010; Proudfoot et al., 2011, 2012).

### POTENTIAL MECHANISMS OF PHYSICAL ACTIVITY AND BIPOLAR DISORDER

The association of physical activity and bipolar disorder might be better understood if the mechanistic pathways could be clarified (Table 2). This next section will examine the current theories on how exercise may impact bipolar disorder.

**TABLE 2:** **Summary of mechanisms between exercise and bipolar disorder**.

**Mechanism**	**Process**	**Implications for bipolar disorder**
Neurogenesis	Pleiotropic, thought to increase neuroplasticity, neurotransmission function, regulation of growth	Improved somatic and psychiatric health for patients with bipolar disorder
Epigenetics	Facilitation of differential gene expression	“Good stress” of physical exercise could increase BDNF expression to improve neurogenesis
Endorphins	Exercise releases endogenous opiates that enhance mood	Improved mood, amelioration of mood symptoms, potential double-edged sword for patients experiencing mania

#### Neurogenesis

One likely mechanism for the benefits observed in bipolar disorder is the causal relationship of increased physical activity and neurogenesis. Exercise is likely a pleiotropic intervention that engages a wide spectrum of neurobiological systems implicated in neurogenesis and neuroplasticity, neurotransmission function, metabolism, immune-inflammatory function and cellular respiration. Data suggest that structured exercise exerts a salutary effect on these interacting networks and therefore, are capable of improving psychiatric and somatic health in bipolar disorder (Alsuwaidan et al., 2009). Several studies have highlighted the beneficial effects of exercise on brain health, with a particular focus on the relationship between voluntary exercise and increased growth factors resulting in neurogenesis, metabolism, vascular function and neurodegeneration and alleviation of depressed mood (Ernst et al., 2006; Zheng et al., 2006; Cotman et al., 2007; Marais et al., 2009; Kucyi et al., 2010; Berk et al., 2011). Exercise is thought to ensure improved brain function by increasing synaptic plasticity, regulation of growth factors and reduction of peripheral and central risk factors (Cotman et al., 2007).

One of the best candidates for explaining the relationship of exercise with neurogenesis—to ultimately improve outcomes in bipolar disorder—is BDNF (Sylvia et al., 2010). Up-regulation of hippocampal BDNF is a well-documented result of chronic antidepressant administration as well as one of the most robust, sustained and consistently demonstrated changes as a result of exercise (Duman et al., 2008). BDNF is a member of the neurotrophin family and promotes neuronal survival and regeneration and is implicated as a biomarker of disease activity in psychiatric disorders (Frey et al., 2013; Fernandes et al., 2014). This past year, researchers further clarified the exercise-BDNF pathway. Specifically, they found that FNDC5, a recently discovered muscle protein, is elevated by endurance exercise in the hippocampus of mice and that peroxisome proliferator-activated receptors (PGC-1α) and FNDC5 regulate BDNF expression in the brain (Wrann et al., 2013). This model supposes that exercise leads to increased transcription of PGC-1α and up-regulation of Erra α (a nuclear receptor estrogen-related receptor) which is necessary to induce FNDC5 gene expression and ultimately, increase BDNF. Of note, the upregulation of BDNF through exercise shares a similar pathway to that of antidepressants which could theoretically lead to exercise triggering potential manic episodes. Supporting this theory, studies of animals have found that exercise may also impact BDNF by increasing serotonin in the frontal cortex and ventral hippocampus, or mimic the SSRI pathway (Eyers and Parker, 1997; Marais et al., 2009).

#### Endorphins

It is also possible that the beneficial effects of exercise on mood may be due to its association with endorphins (Steinberg and Sykes, 1985). This theory proposes that exercise is associated with release of endogenous opiates including α endorphins that improve mood and feelings of well-being. Similarly, the monoamine hypothesis suggests that exercise results in an increase release of the monoamine molecules dopamine, serotonin and norepinephrine that are typically reduced in depression (Pierce et al., 1976). Ernst et al. (2006) also found that an increase in α endorphins, BDNF, vascular endothelial growth factor (VEGF), and serotonin release may account for the relationship exercise and positive outcomes on mood and functioning.

#### Epigenetics

Among hypothesized pathways of exercise and bipolar disorder is epigenetics as exercise may elevate BDNF via these mechanisms (Gomez-Pinilla et al., 2011). Epigenetic mechanisms facilitate differential gene expression, which are subject to environmental influence and have been implicated in the pathophysiology of bipolar disorder (Rao et al., 2012; Banigan et al., 2013; Gamazon et al., 2013; Niculescu, 2013). These mechanisms may mediate some of the physiological impacts of exercise on body tissues (McGee and Hargreaves, 2011; Barres et al., 2012). Epigenetic gene expression alterations induced by “eustress” or “good stress” of physical exercise appear to have beneficial effects (Sanchis-Gomar et al., 2012). For example, BDNF methylation has been implicated in several psychiatric disorders, including bipolar disorder (Ikegame et al., 2013). Taken together, it seems plausible that some of the beneficial associations between exercise and outcome of bipolar disorders are mediated by epigenetic mechanisms.

#### Other pathways

Exercise increases mitochondrial energy generation (Boushel et al., 2014), and it is known that in depression, particularly in bipolar depression that there is decreased mitochondrial bioenergetics capacity (de Sousa et al., 2014). Similarly, in bipolar disorder there is increased inflammation and oxidative stress (Berk et al., 2011, 2013; Moylan et al., 2014), and exercise reduces both markers of systemic inflammation and oxidative stress (Jatoi, 2013). Exercise reduces cortisol, long known as elevated in depression (Rezaee et al., 2014). Lastly, other factors such as adipokines are implicated as depression biomarkers (Carvalho et al., 2014), and the effects of exercise may be mediated by adipokines such as adiponectin (Yau et al., 2014).

## CONCLUSION

Despite the promise of exercise to meet the physical and mental health needs of individuals with bipolar disorder, there is a dearth of literature investigating the role of exercise for bipolar disorder. Furthermore, the current literature is riddled with limitations, such as small samples, heterogeneous treatment groups, no control groups, no distinction between types of exercise (structured exercise programs vs. lifestyle physical activity), clear definitions of the amount (duration, frequency and intensity) of exercise, as well as empirical data with regards to mood-state-dependent effects of exercise for individuals specifically with bipolar disorder. Finally, high attrition rates are often observed in research with this population, potentially leading to biased results. As a result, there is limited information to guide clinicians as to the appropriate intensity, frequency and duration of exercise for people with bipolar disorder and it is thus impossible to give bipolar-specific guidelines for exercise (Barbour et al., 2007; Wright et al., 2012).

There are promising data that exercise may be a viable and effective strategy to deal with the depressive phase of bipolar disorder, but further research is needed to determine the recommended intensity, duration and frequency of exercise programs. It is also necessary for researchers in the future to differentiate between physical activity as leisure-based pursuits, occupational and incidental activity, and more structured, planned, and voluntary exercise. In short, due to the unique problems that patients with bipolar disorder face, such as pharmacotherapy needs, often extreme fluctuations in mood symptoms, and a high comorbidity rate, it is imperative that more research be conducted in this arena so that we can better tailor adjunct lifestyle programs for them.

## AUTHOR CONTRIBUTIONS

DT and MG conducted the literature searches; all authors contributed to data interpretation, manuscript preparation and final approval.

### Conflict of Interest Statement

No funding has been received to support this study. Ajeet B. Singh is a casual speaker for Servier Australia, Pfizer Australia, and Lundbeck Australia. Julie A. Pasco has received grant/research support from the NHMRC, Perpetual, Amgen (Europe) GmBH, and BUPA Foundation and has received speaker fees from Amgen and Sanofi Aventis. Michael Berk is supported by a NHMRC Senior Principal Research Fellowship 1059660 and has received Grant/Research Support from the NIH, Cooperative Research Centre, Simons Autism Foundation, Cancer Council of Victoria, Stanley Medical Research Foundation, MBF, NHMRC, Beyond Blue, Rotary Health, Geelong Medical Research Foundation, Bristol Myers Squibb, Eli Lilly, Glaxo SmithKline, Meat and Livestock Board, Organon, Novartis, Mayne Pharma, Servier and Woolworths, has been a speaker for Astra Zeneca, Bristol Myers Squibb, Eli Lilly, Glaxo SmithKline, Janssen Cilag, Lundbeck, Merck, Pfizer, Sanofi Synthelabo, Servier, Solvay and Wyeth, and served as a consultant to Astra Zeneca, Bioadvantex, Bristol Myers Squibb, Eli Lilly, Glaxo SmithKline, Janssen Cilag, Lundbeck Merck and Servier. Louisa Sylvia was a shareholder in Concordant Rater Systems and serves as a consultant for United Biosource Corporation, Clinical Trials Network and Institute, Clintara. She receives royalties from New Harbinger.
